# Supplementary material for: Antibody toolkit reveals N-terminally ubiquitinated substrates of UBE2W
Source: Nat Commun. 2021 Jul 29;12:4608. doi: 10.1038/s41467-021-24669-6 (PMC8322077; doi:10.1038/s41467-021-24669-6)
Supplement: Supplementary file 1 — Supplementary Information [file 41467_2021_24669_MOESM1_ESM.pdf]

Supplementary Information for

Antibody toolkit reveals N-terminally ubiquitinated substrates of UBE2W

Christopher W. Davies[1,8], Simon E. Vidal[2,8], Lilian Phu[3,8], Jawahar Sudhamsu[4], Trent B. Hinkle[3], Scott Chan-Rosenberg[2], Frances-Rose Schumacher[3], Yi Jimmy Zeng[3], Carsten Schwerdtfeger[5], Andrew S. Peterson[6], Jennie R. Lill[3], Christopher M. Rose[3], Andrey S Shaw[7], Ingrid E. Wertz[2]*, Donald S. Kirkpatrick[3]*, James T. Koerber[1]*

*Correspondence to ingrid.wertz@bms.com (I.E.W.), dkirkpatrick@interlinetx.com (D.S.K.), or koerberj@gene.com (J.T.K.).

This PDF file includes:

Supplementary Tables 1-3

Supplementary Figures 1-7

Other supplementary materials for this manuscript include the following:

Supplementary Data 1 and 2

Supplementary Information

Supplementary Table 1. Data collection and refinement statistics for the 1C7 Fab GGM peptide co-crystal structure. Values in parentheses are for highest-resolution shell.

	1C7 Fab-GGM peptide complex
Data collection	
Space group	P 4_3 2_1 2
Cell dimensions	
a, b, c (Å)	163.80, 163.80, 127.47
α, β, γ	90, 90, 90
Resolution (Å)	48.08-2.85 (2.92-2.85)
R_{pim}	0.039 (0.477)
I / σ (I)	22.40 (1.46)
Completeness (%)	99.5 (98.7)
Redundancy	6.6 (6.2)
$CC_{1/2}$	0.999 (0.806)
CC*	0.998 (0.945)
Refinement	
Resolution (Å)	2.85
No. of reflections	40874 (2701)
R_{work} / R_{free}	0.211 / 0.260
No. of atoms	6568
Protein	6479
Ligand/ion	50
Water	39

Average B-factors	78.0
Protein	78.20
Ligand/ion	106.3
Water	73.1
R.m.s deviations	
Bond lengths (Å)	0.0091
Bond angles (°)	1.114

Supplementary Table 2. Unique and shared internal GGX sequences from anti-GGX mAbs

mAb	Unique sequences	Shared sequences
1C7	26	16
2E9	41	14
2H2	47	20
2B12	8	8

Supplementary Table 3. List of primers used to amplify the heavy and light chain repertoire from rabbits

BssHII.Rab.VH1	ATTGCTACAAATGCCTATGCAGCGCGC CAG GAG CAG CTG AAG GAG TCC
BssHII.Rab.VH2/14	ATTGCTACAAATGCCTATGCAGCGCGC CAG GAG CAG CTG GAG GAG TCC
BssHII.Rab.VH3	ATTGCTACAAATGCCTATGCAGCGCGC CAG GAG CAG CTG GTG GAG TCC
BssHII.Rab.VH4	ATTGCTACAAATGCCTATGCAGCGCGC CAG TCG GTG AAG GAG TCC GAG
BssHII.Rab.VH5	ATTGCTACAAATGCCTATGCAGCGCGC CAG TCG STG GAG GAG TCC AGG
BssHII.Rab.VH6	ATTGCTACAAATGCCTATGCAGCGCGC CAG TCG STG GAG GAG TCC GGG
BssHII.Rab.VH7	ATTGCTACAAATGCCTATGCAGCGCGC CAG SAG CAG CTG GAG GAG TCC
BssHII.Rab.VH8	ATTGCTACAAATGCCTATGCAGCGCGC CAG TCG YTG GRG GAR TYC RGG
BssHII.Rab.VH9	ATTGCTACAAATGCCTATGCAGCGCGC CAG RAG CAG CTG RTG GAG TCC
BssHII.Rab.VH10	ATTGCTACAAATGCCTATGCAGCGCGC CAG CAG CTG AAG GAG TCC GGA
BssHII.Rab.VH11	ATTGCTACAAATGCCTATGCAGCGCGC CAG GAG CAG CAG AAG GAG TCC
BssHII.Rab.VH12	ATTGCTACAAATGCCTATGCAGCGCGC CAG ACA GTG AAG GAG TCC GAG
BssHII.Rab.VH13	ATTGCTACAAATGCCTATGCAGCGCGC GAG GAT CAG CTA GTG GAG TCC
GS.Rab.JH1/5/6	ACCACCGCTGCCGCCGCCGCCTGATCCTCCTCCTCC TGA GGA GAC GGT GAC CAG GGT
GS.Rab.JH2/3/4	ACCACCGCTGCCGCCGCCGCCTGATCCTCCTCCTCC TGA AGA GAC GGT GAC CAG GGT
GS.Rab.VK1	GGATCAGGCGGCGGCGGCAGCGGTGGTGGTGGTTCG GAC CCT ATG CTG ACC CAG ACT
GS.Rab.VK2	GGATCAGGCGGCGGCGGCAGCGGTGGTGGTGGTTCG GAC CCT GTG CTG ACC CAG ACT
GS.Rab.VK3	GGATCAGGCGGCGGCGGCAGCGGTGGTGGTGGTTCG GAC CCT RTG CTG ACC CAG ACW CC
GS.Rab.VK4	GGATCAGGCGGCGGCGGCAGCGGTGGTGGTGGTTCG GAT GTY GTG ATG ACC CAG ACT
GS.Rab.VK5	GGATCAGGCGGCGGCGGCAGCGGTGGTGGTGGTTCG GAT GKC GTG ATG ACC CAG ACT
GS.Rab.VK6	GGATCAGGCGGCGGCGGCAGCGGTGGTGGTGGTTCG GCA GCC GTG CTG ACC CAG ACA
GS.Rab.VK7	GGATCAGGCGGCGGCGGCAGCGGTGGTGGTGGTTCG TAT GTC ATG ATG ACC CAG ACT
GS.Rab.VK8	GGATCAGGCGGCGGCGGCAGCGGTGGTGGTGGTTCG GCC CTT GTG ATG ACC CAG ACT
GS.Rab.VK9	GGATCAGGCGGCGGCGGCAGCGGTGGTGGTGGTTCG GCC ATC RAW ATG ACC CAG ACT
GS.Rab.VK10	GGATCAGGCGGCGGCGGCAGCGGTGGTGGTGGTTCG GCC CAA GTG CTG ACC CAG ACT
GS.Rab.VK11	GGATCAGGCGGCGGCGGCAGCGGTGGTGGTGGTTCG GAC MYT GTG MTG ACC CAG ACT
GS.Rab.VK12	GGATCAGGCGGCGGCGGCAGCGGTGGTGGTGGTTCG GCM GYC GTG MTG ACC CAG ACT

SfiI.Rab.JK1	GTGATGGTGGTGAGCCTTGGCCCCGGTGGCCGC TTTGATTTCYACMTTGGTGCC
SfiI.Rab.JK2	GTGATGGTGGTGAGCCTTGGCCCCGGTGGCCGC TTYGACSACCACCTYGGTCCC
SfiI.Rab.JK3	GTGATGGTGGTGAGCCTTGGCCCCGGTGGCCGC TAGGATCTCCAGCTCGGTCCC
SfiI.Rab.JK4	GTGATGGTGGTGAGCCTTGGCCCCGGTGGCCGC TTTGATTTCCAGTTTGGTCCC
SfiI.Rab.JK5	GTGATGGTGGTGAGCCTTGGCCCCGGTGGCCGC TYKRATCTCCACCATGGTCCC
SfiI.Rab.JK6	GTGATGGTGGTGAGCCTTGGCCCCGGTGGCCGC TTTGATCTCCASCTTGGTCYC
SfiI.Rab.JK7	GTGATGGTGGTGAGCCTTGGCCCCGGTGGCCGC TTTGATCTCCAGCTTGGTTCC

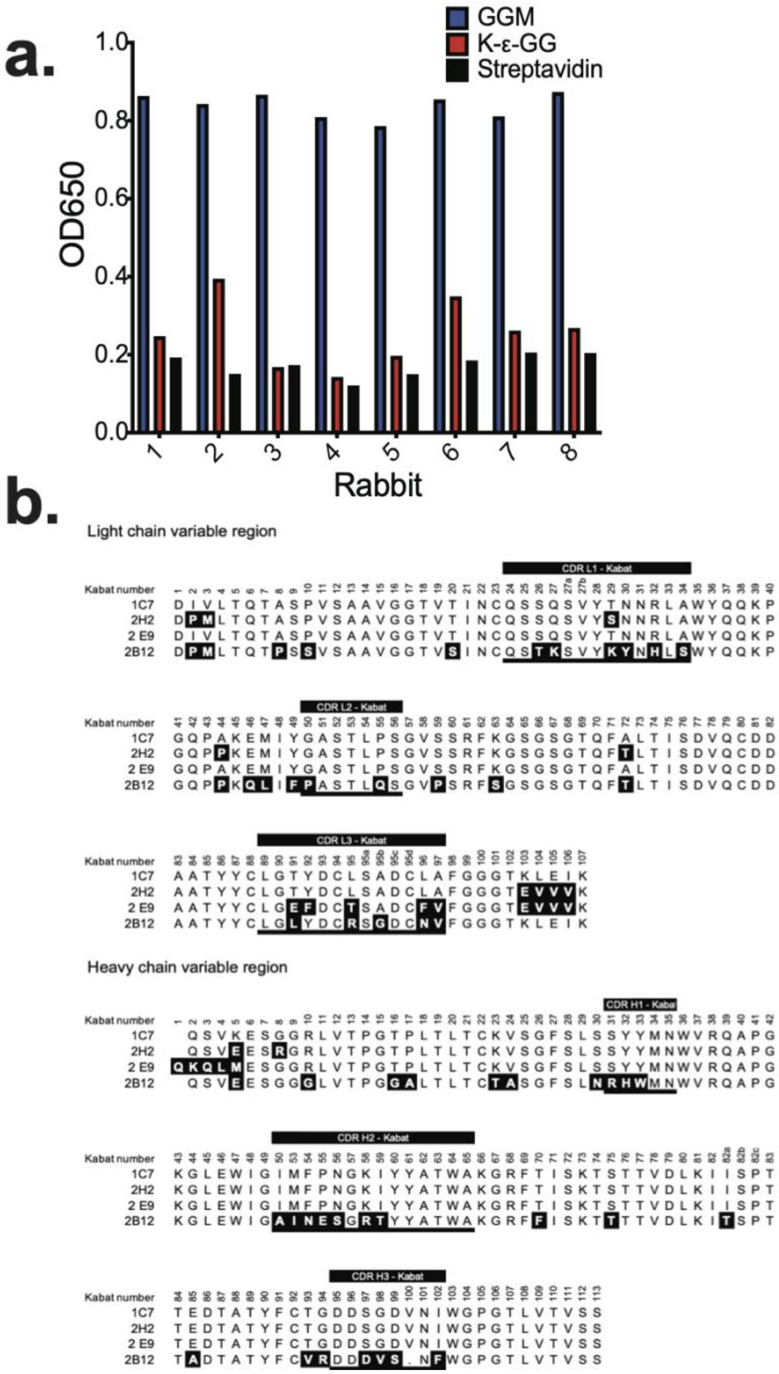

Supplementary Figure 1. **pAb characterization and sequence alignment of anti-GGX antibodies.** (a) ELISA characterization of pAb response from each of the eight rabbits against the GGM (blue) and K-ε-GG (red) peptides, with streptavidin as control.

Source data are provided as a Source Data file. (b) Sequence alignment of the LC and HC sequences of the four anti-GGX mAbs.

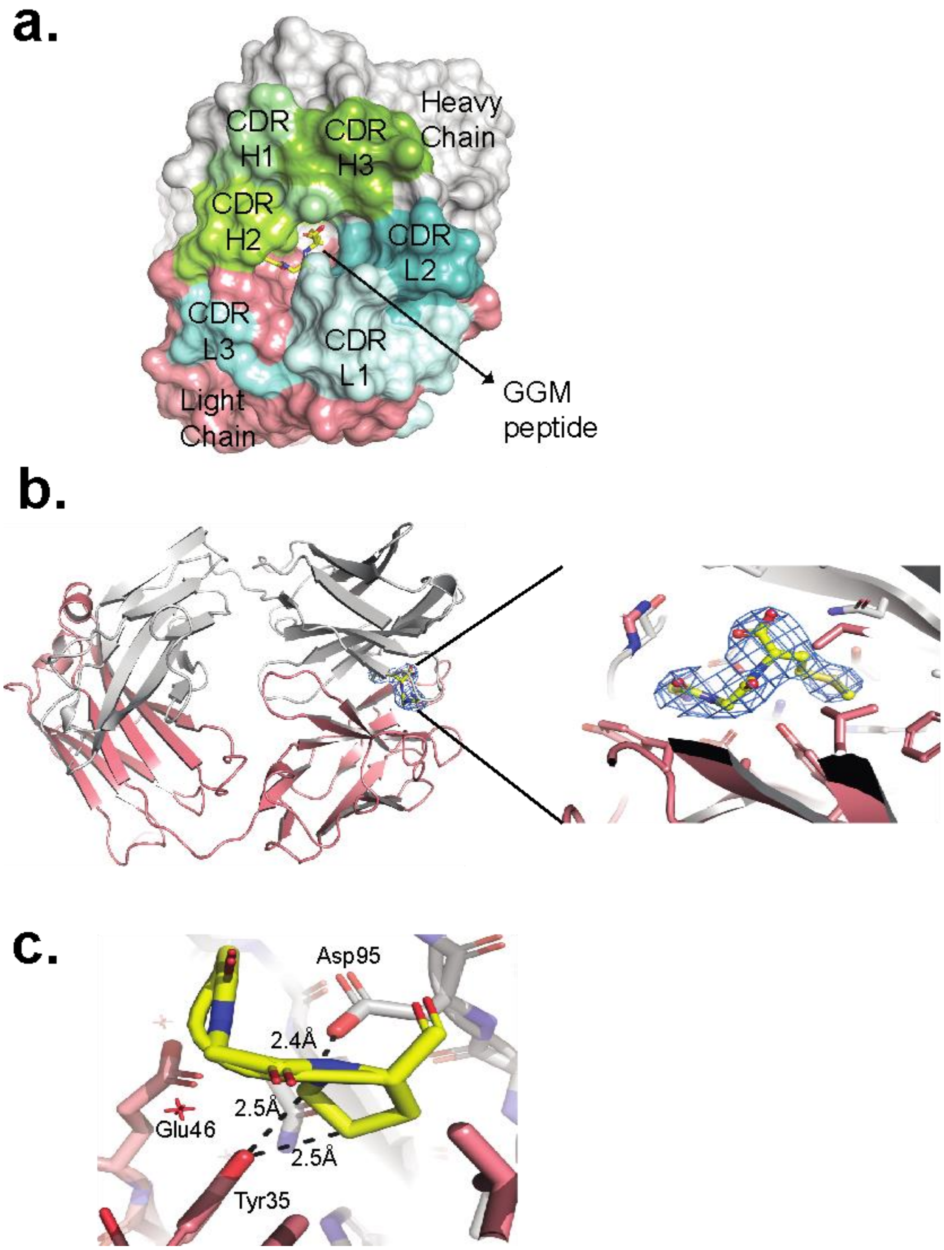

Supplementary Figure 2. **Global structural analysis of GGM peptide bound to anti-GGX Fab.** (a) Surface representation of 1C7 Fab bound to GGM peptide. (c) Cartoon representation of the 1C7 Fab bound to GGM peptide enveloped within the electron density mesh (2Fo-Fc), contoured at 1σ, showing that the peptide is well-defined within the structure. (c) GGP peptide (yellow) modelled into the structure of 1C7 Fab shows multiple clashes with HC Asp95 and LC Tyr35.

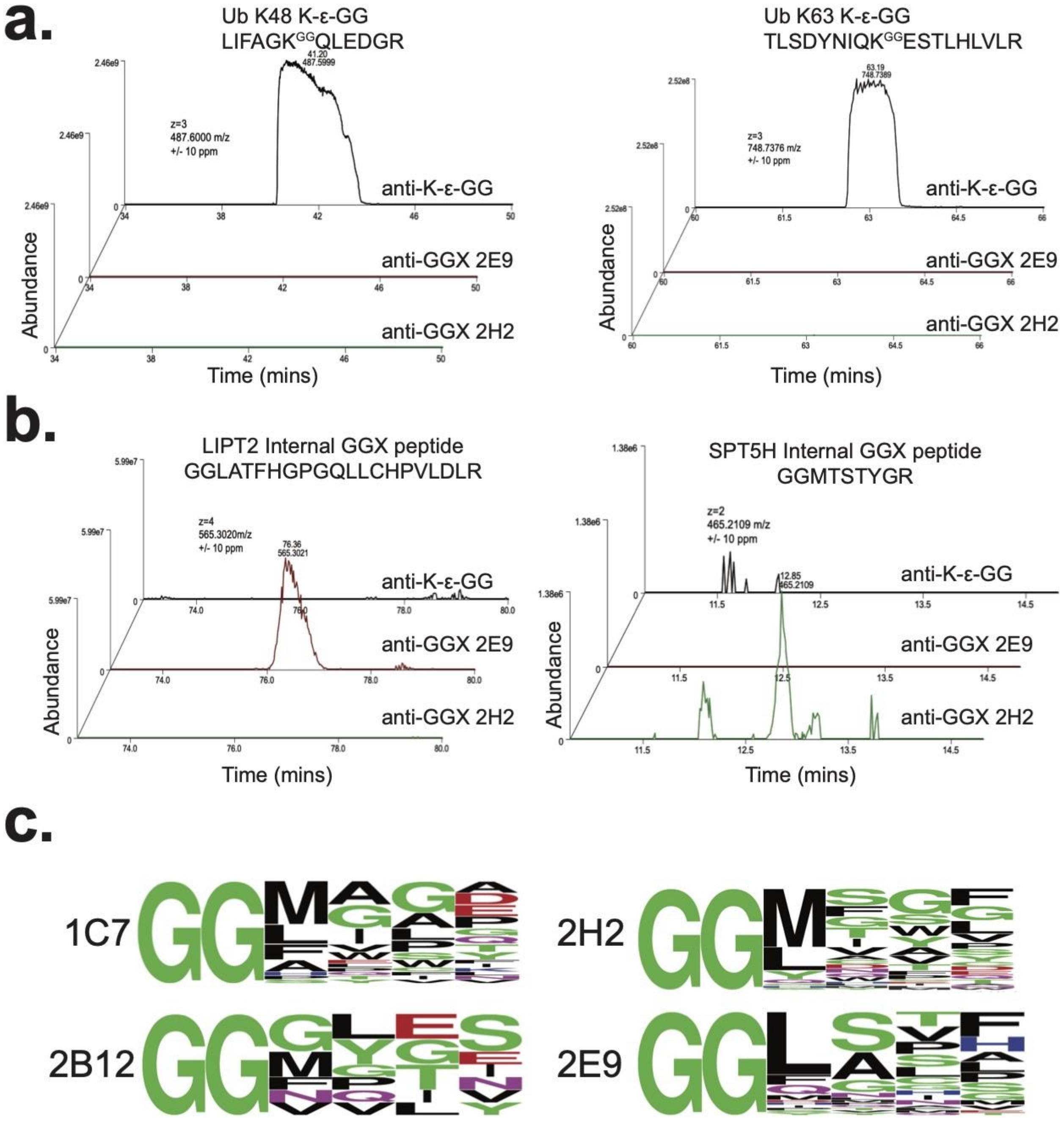

Supplementary Figure 3. **MS validation that anti-GGX mAbs selectively IP GGX peptides.** (a) Extracted ion chromatograms (+/- 10 ppm) for K48 and K63 K-ε-GG polyubiquitin chain linkage peptides LIFAGK^{GG}QLEDGR and TLSDYNIQK^{GG}ESTLHLVLR in anti-K-ε-GG, anti-GGX 2E9, and anti-GGX 2H2

immunoaffinity enrichment MS experiments. (b) Extracted ion chromatograms (+/- 10 ppm) for internal GGX peptides GGLATFHGPGQLLCHPVLDLR and GGMTSTYGR in anti-K-ε-GG, anti-GGX 2E9, and anti-GGX 2H2 immunoaffinity enrichment MS experiments. (c) WebLogos representing the sequence diversity of internal GGX peptides enriched by each of the anti-GGX mAbs.

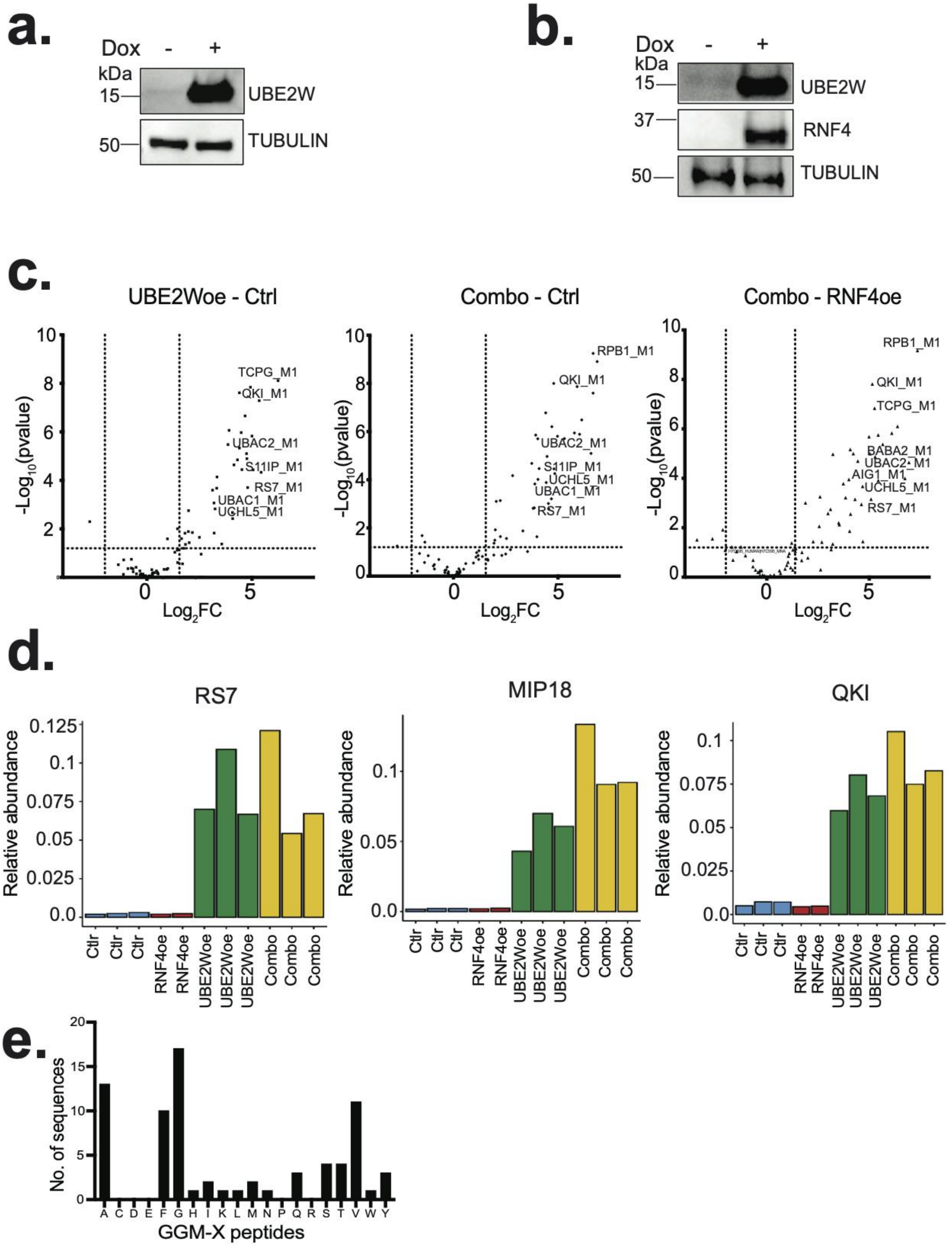

a.
Dox - +
kDa
15
UBE2W
50
TUBULIN
b.
Dox - +
kDa
15
UBE2W
37
RNF4
50
TUBULIN
c.
UBE2Woe - Ctrl
Combo - Ctrl
Combo - RNF4oe
-Log10(pvalue)
Log2FC
TCPG_M1
QKI_M1
UBAC2_M1
S11IP_M1
RS7_M1
UBAC1_M1
UCHL5_M1
RPB1_M1
BABA2_M1
AIG1_M1
d.
RS7
MIP18
QKI
Relative abundance
Ctlr
RNF4oe
UBE2Woe
Combo
e.
No. of sequences
A C D E F G H I K L M N P Q R S T V W Y
GGM-X peptides

Supplementary Figure 4. **UBE2W overexpression reveals substrates.** (a) Western blot of stable doxycycline-inducible *UBE2W* HEK293 cells at 24 hrs post doxycycline treatment. Results are representative of independent replicates (n=3). (b) Western blot of stable Dox -inducible *UBE2W/RNF4* HEK293 cells at 24 hrs post Dox treatment. Results are representative of independent replicates (n=3). (c) Volcano plots showing differential N-terminal protein ubiquitination data for "UBE2Woe versus Control", "Combo versus RNF4oe", and "Combo versus Control" conditions in label free GGX-MS experiment. Each data point represents one protein. Cutoffs are displayed by dashed lines at absolute $\log_2$ fold change >1.0 and $-\log_{10}$ *P* value >1.3 ($P < 0.05$). R package MSstats was used for statistical analysis to perform differential abundance analysis. MSstats estimated log2(fold change) and the standard error by linear mixed effect model for each protein. To test two-sided null hypothesis of no changes in abundance, the model-based test statistics were compared to the Student t-test distribution with the degrees of freedom appropriate for each protein and each dataset. (d) Protein bar plots displaying relative N-terminal ubiquitination abundances for individual TMT-11plex channels corresponding to biological replicates (n=3). (e) Analysis of the second position of immunoaffinity enriched UBE2W substrates indicates a preferential enrichment for peptides that contain glycine, alanine, valine, or phenylalanine after the initiator methionine. Source data are provided as a Source Data file.

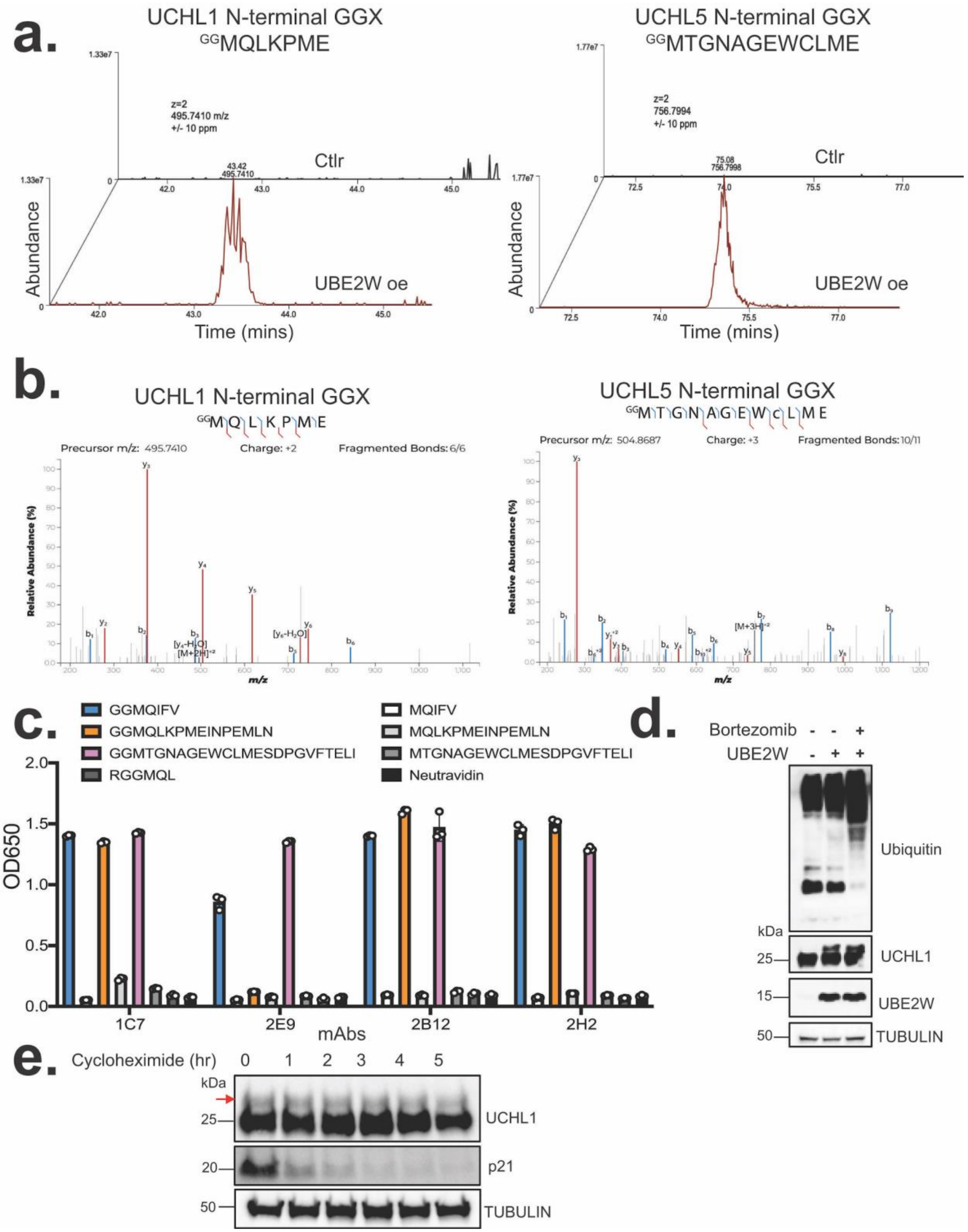
a.
UCHL1 N-terminal GGX
GGMQLKPME
z=2
495.7410 m/z
+/- 10 ppm
Ctlr
UBE2W oe
Abundance
Time (mins)
UCHL5 N-terminal GGX
GGMTGNAGEWCLME
z=2
756.7994
+/- 10 ppm
b.
UCHL1 N-terminal GGX
Precursor m/z: 495.7410
Charge: +2
Fragmented Bonds: 6/6
Relative Abundance (%)
m/z
UCHL5 N-terminal GGX
Precursor m/z: 504.8687
Charge: +3
Fragmented Bonds: 10/11
c.
GGMQIFV
GGMQLKPMEINPEMLN
GGMTGNAGEWCLMESDPGVFTELI
RGGMQL
MQIFV
MQLKPMEINPEMLN
MTGNAGEWCLMESDPGVFTELI
Neutravidin
OD650
1C7
2E9
2B12
2H2
mAbs
d.
Bortezomib
UBE2W
Ubiquitin
kDa
UCHL1
UBE2W
TUBULIN
e.
Cycloheximide (hr)
kDa
UCHL1
p21
TUBULIN

Supplementary Figure 5. **UCHL1 and UCHL5 are substrates of UBE2W, but N-terminal ubiquitination is not a signal for proteasomal degradation.** (a) Extracted ion chromatograms (+/- 10 ppm) for N-terminal semi-tryptic GGX peptides ^{GG}MQLKPME and ^{GG}MTGNAGEWCLME of UCHL1 and UCHL5, respectively, in Control and UBE2Woe conditions from GGX- IAP-LC-MS/MS experiment. (b) MS/MS spectra identifications of N-terminal semi-tryptic GGX modified peptides ^{GG}MQLKPME (doubly charged, 495.7406 m/z) and ^{GG}MTGNAGEWCLME (doubly charged, 756.7994 m/z). Detected b- and y- ions highlighted in blue and red, respectively. (c) ELISA characterization of the xGGM mAbs against the N-terminal tryptic peptides that correspond to the free N-terminus of ubiquitin (MQIFV), UCHL1 (MQLKPMEINPEMLN) and UCHL5 (MTGNAGEWCLMESDPGVFTELI) and the GG-modified N-terminus of these proteins. The GG-modified peptides are shown in blue, orange, or pink, whereas, the free N-terminal peptides and R-GG control peptide are shown in shades of gray, black, or white. Results are representative of biological replicates (n=3). Data are presented as mean +/- SD. (d) Western blots of doxycycline -inducible *UBE2W/RNF4* HEK293 cells at 24 hrs post doxycycline treatment. Cells were additionally treated with proteasome inhibitor Bortezomib (Btz) (10 μM, 2 h) before cell harvest. Results are representative of 3 independent experiments. (e) Western blots against UCHL1, p21 and tubulin of Dox-inducible *UBE2W/RNF4* HEK293 cells at 24 hrs post Dox treatment. Cells were additionally treated with cycloheximide (10 μg/ml) for the indicated times before cell harvest. Results are representative of 3 independent experiments. Source data are provided as a Source Data file.

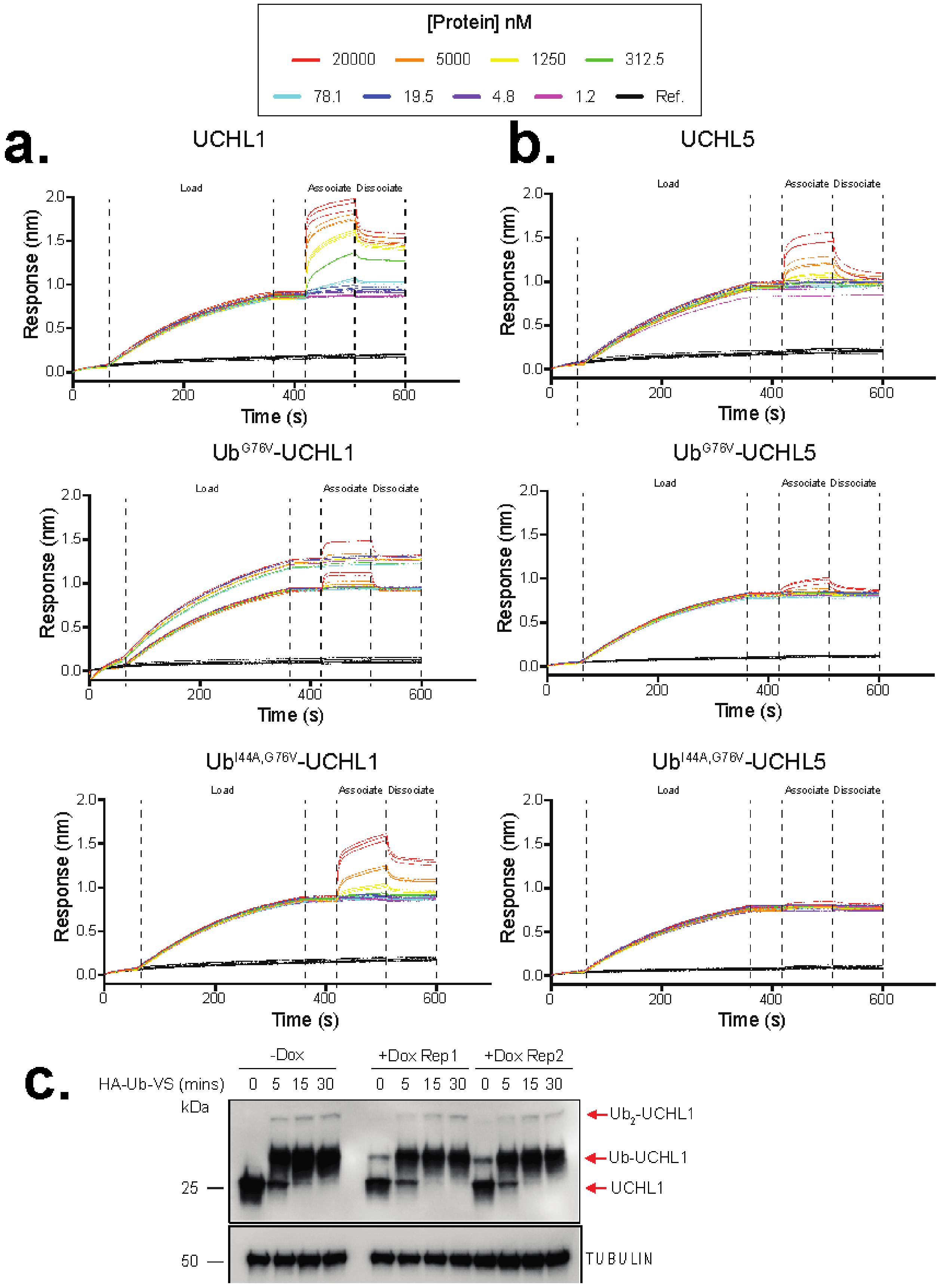

[Protein] nM
20000
5000
1250
312.5
78.1
19.5
4.8
1.2
Ref.
a.
UCHL1
Ub^{G76V}-UCHL1
Ub^{I44A,G76V}-UCHL1
b.
UCHL5
Ub^{G76V}-UCHL5
Ub^{I44A,G76V}-UCHL5
Load
Associate
Dissociate
Response (nm)
Time (s)
c.
-Dox
+Dox Rep1
+Dox Rep2
HA-Ub-VS (mins)
0 5 15 30
kDa
25
50
Ub$_2$-UCHL1
Ub-UCHL1
UCHL1
TUBULIN

Supplementary Figure 6. **N-terminal ubiquitination blocks Ub binding to UCHL1 and UCHL5.** (a) Representative sensorgrams showing the binding of Ub to wild-type UCHL1, the N-terminally ubiquitinated mimetic (Ub^{G76V}-UCHL1), or the N-terminally ubiquitinated mimetic with reduced ubiquitin binding ($Ub^{I44A,G76V}$-UCHL1). (b) Representative sensorgrams showing the binding of Ub to wild-type UCHL5, the N-terminally ubiquitinated mimetic (Ub^{G76V}-UCHL5), or the N-terminally ubiquitinated mimetic with reduced ubiquitin binding ($Ub^{I44A,G76V}$-UCHL5). (c) Activity assay with whole-cell extracts. UCHL1 was allowed to react with the suicide probe Ubiquitin-Vinyl Sulfone (Ub-VS) without UBE2W expression (-Dox), and with UBE2W overexpression (+Dox) for the indicated time points. The red arrow indicates the band associated with the indicated proteins. Results are from 2 independent replicates for +Dox. Source data are provided as a Source Data file.

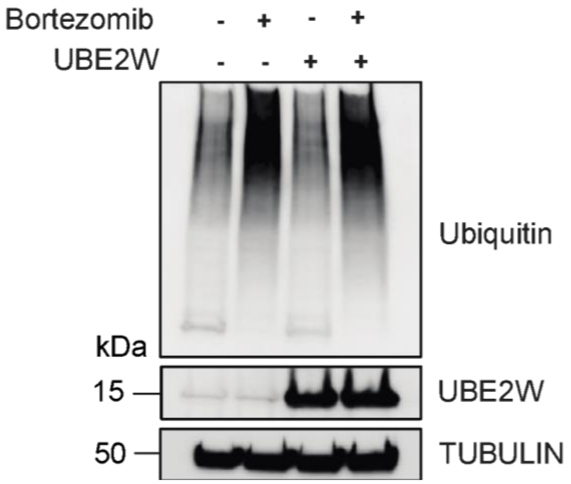

Supplementary Figure 7. **Western blot validation of proteasome inhibition by Bortezomib.** Western blots of doxycycline -inducible *UBE2W/RNF4* HEK293 cells at 24 hrs post doxycycline treatment. Cells were additionally treated with proteasome inhibitor (Btz) (10 μM, 2 h) before cell harvest. Results are representative of 3 independent experiments. Source data are provided as a Source Data file.
