# Supplementary material for: Antibody toolkit reveals N-terminally ubiquitinated substrates of UBE2W
Source: Nat Commun. 2021 Jul 29;12:4608. doi: 10.1038/s41467-021-24669-6 (PMC8322077; doi:10.1038/s41467-021-24669-6)
Supplement: Supplementary file 3 — Description of Additional Supplementary Files [file 41467_2021_24669_MOESM3_ESM.docx]

**Description of Additional Supplementary Files**

**File Name:** Supplementary Data 1

**Description:** List of identified N-terminally ubiquitinated substrates

**File Name:** Supplementary Data 2

**Description:** List of identified N-terminally ubiquitinated substrates upon proteasome inhibition
