# Supplementary material for: Antibody toolkit reveals N-terminally ubiquitinated substrates of UBE2W
Source: Nat Commun. 2021 Jul 29;12:4608. doi: 10.1038/s41467-021-24669-6 (PMC8322077; doi:10.1038/s41467-021-24669-6)
Supplement: Supplementary file 6 — Reporting Summary [file 41467_2021_24669_MOESM6_ESM.pdf]

nature research

Corresponding author(s): Ingrid E. Wertz, Donald S. Kirkpatrick, James T. Koerber

Last updated by author(s): 2021/06/22

Reporting Summary

Nature Research wishes to improve the reproducibility of the work that we publish. This form provides structure for consistency and transparency in reporting. For further information on Nature Research policies, see our Editorial Policies and the Editorial Policy Checklist.

Statistics

For all statistical analyses, confirm that the following items are present in the figure legend, table legend, main text, or Methods section.

n/a	Confirmed	
☐	☒	The exact sample size (*n*) for each experimental group/condition, given as a discrete number and unit of measurement
☐	☒	A statement on whether measurements were taken from distinct samples or whether the same sample was measured repeatedly
☐	☒	The statistical test(s) used AND whether they are one- or two-sided<br>*Only common tests should be described solely by name; describe more complex techniques in the Methods section.*
☒	☐	A description of all covariates tested
☒	☐	A description of any assumptions or corrections, such as tests of normality and adjustment for multiple comparisons
☐	☒	A full description of the statistical parameters including central tendency (e.g. means) or other basic estimates (e.g. regression coefficient) AND variation (e.g. standard deviation) or associated estimates of uncertainty (e.g. confidence intervals)
☐	☒	For null hypothesis testing, the test statistic (e.g. *F*, *t*, *r*) with confidence intervals, effect sizes, degrees of freedom and *P* value noted<br>*Give P values as exact values whenever suitable.*
☒	☐	For Bayesian analysis, information on the choice of priors and Markov chain Monte Carlo settings
☒	☐	For hierarchical and complex designs, identification of the appropriate level for tests and full reporting of outcomes
☐	☒	Estimates of effect sizes (e.g. Cohen's *d*, Pearson's *r*), indicating how they were calculated

Our web collection on statistics for biologists contains articles on many of the points above.

Software and code

Policy information about availability of computer code

Data collection	HKL2000
Data analysis	Coot v0.8.9, Phenix v 1.12, Mascot, MSstatsTMTv1.6.3, MSstats v3.20.0, Prism v9.1.2

For manuscripts utilizing custom algorithms or software that are central to the research but not yet described in published literature, software must be made available to editors and reviewers. We strongly encourage code deposition in a community repository (e.g. GitHub). See the Nature Research guidelines for submitting code & software for further information.

Data

Policy information about availability of data

All manuscripts must include a data availability statement. This statement should provide the following information, where applicable:

- Accession codes, unique identifiers, or web links for publicly available datasets
- A list of figures that have associated raw data
- A description of any restrictions on data availability

An existing rabbit Fab structure was used as a model for molecular replacement [https://doi.org/doi:10.2210/pdb4ztp/pdb]. The crystal structure has been deposited in the protein databank (PDB) with the PDB accession code 7MFR [https://doi.org/doi:10.2210/pdb7mfr/pdb]. The mass spectrometry raw files have been uploaded to the UCSD MassIVE repository will accession code MSV000086537 [https://massive.ucsd.edu/ProteoSAFe/dataset.jsp?accession=MSV000086537]. The Uniprot [https://www.uniprot.org] and TrEMBL databases [https://www.uniprot.org/uniprot/?query=reviewed:no] were used for the mass spectrometry analysis. Source data are provided with this paper.

Field-specific reporting

Please select the one below that is the best fit for your research. If you are not sure, read the appropriate sections before making your selection.

[x] Life sciences [ ] Behavioural & social sciences [ ] Ecological, evolutionary & environmental sciences

For a reference copy of the document with all sections, see nature.com/documents/nr-reporting-summary-flat.pdf

Life sciences study design

All studies must disclose on these points even when the disclosure is negative.

Sample size	Three biological replicates were performed, which was sufficient to show data reproducibility. No sample-size calculations were performed.
Data exclusions	No data was excluded from analysis.
Replication	Three biological replicates were done for peptide-antibody ELISAs. Two or three biological replicates were used for all mass spec experiments. In vitro ubiquitination assays were performed with 3 independent runs. Three technical replicates were performed for the BLI experiment. The Ub-Rh110 enzymatic assays were performed with 2 independent runs. The Ub vinyl sulfone assay was performed in biological triplicate. All Western blots were performed 2 or 3 times, as specified in the text and figure captions.
Randomization	Randomization was not needed for this study, since samples were not divided into groups.
Blinding	No blinding was performed since group analysis was not performed.

Reporting for specific materials, systems and methods

We require information from authors about some types of materials, experimental systems and methods used in many studies. Here, indicate whether each material, system or method listed is relevant to your study. If you are not sure if a list item applies to your research, read the appropriate section before selecting a response.

Materials & experimental systems

n/a	Involved in the study	
[ ]	[x]	Antibodies
[ ]	[x]	Eukaryotic cell lines
[x]	[ ]	Palaeontology and archaeology
[ ]	[x]	Animals and other organisms
[x]	[ ]	Human research participants
[x]	[ ]	Clinical data
[x]	[ ]	Dual use research of concern

Methods

n/a	Involved in the study	
[x]	[ ]	ChIP-seq
[x]	[ ]	Flow cytometry
[x]	[ ]	MRI-based neuroimaging

Antibodies

Antibodies used

Rabbit anti-beta Tubulin (Abcam, Cat# ab6046, Lot#GR3331866-2)
Rabbit anti-UBE2W (Thermo Fisher, cat#PA5-67547, Lot#VA2920556)
Rabbit anti-HA tag (Cell Signaling Technology, Cat#3724S, Lot#9)
Mouse anti-RNF4 (Thermo Fisher, Cat#MA527423, Lot#UD2752046)
Rabbit anti-p21 [EPR3993] (abcam, Cat#ab109199, Lot#GR3250394-2)
Rabbit anti-UCHL1 (Thermo Fisher cat# HPA005993, Lot#C104414)
Rabbit anti-UCH37 [EPR4896] (abcam, Cat#ab124931, Lot#GR82268-10)
Mouse anti-Ubiquitin (Life Sensors, cat# VU-1, Lot#VU-43653.001)
Goat anti-mouse IgG HRP (Thermo Fisher, cat# 31430, Lot#TE262980)
Goat anti-rabbit IgG (Thermo Fisher, cat#31460, TB260029)
Ubiquitin Remnant Motif (K-e-GG) (D4A7) Rabbit mAb (Cell Signaling Technology, Ref: 06/2017, 3925BF, Lot#9)
Peroxidase AffiniPure Goat anti-rabbit IgG (H+L) (Jackson ImmunoResearch, Code:111-035-144, Lot#145476)
1C7/2E9/2H2/2B12 (Generated by Genentech and described in this paper)

Validation

Antibodies 1C7,2E9,2H2, and 2B12 were validated for specificity by peptide ELISA, crystal structure determination, and by immunoprecipitation coupled with mass spectrometry (IP-MS) as described in this manuscript. Validation of the other antibodies used in this study were done by the manufacturer and the details of these validation efforts are described on the manufacturer's website.

Eukaryotic cell lines

Policy information about cell lines

Cell line source(s)	Expi293F (ThermoFisher Scientific), 293T (Thermo Fisher Scientific), COS-7 (Lonza)
Authentication	None of the cell lines used were authenticated.
Mycoplasma contamination	Negative
Commonly misidentified lines (See ICLAC register)	No commonly misidentified cell lines were used.

Animals and other organisms

Policy information about studies involving animals; ARRIVE guidelines recommended for reporting animal research

Laboratory animals	New Zealand white rabbits, female, 6 months old
Wild animals	n/a
Field-collected samples	n/a
Ethics oversight	All animals used in this study were housed and maintained at Lampire Biological Laboratories (Pipersville, PA) in accordance with American Association of Laboratory Animal Care guidelines. All experimental studies were conducted under protocols approved by the Institutional Animal Care and Use Committee of Lampire and Genentech Lab Animal Research in an Association for Assessment and Accreditation of Laboratory Animal Care International-accredited facility in accordance with the Guide for the Care and Use of Laboratory Animals and applicable laws and regulations.

Note that full information on the approval of the study protocol must also be provided in the manuscript.
